# Angularly selective thermal emitters for deep subfreezing daytime radiative cooling

**DOI:** 10.1515/nanoph-2022-0032

**Published:** 2022-08-05

**Authors:** Sandeep Kumar Chamoli, Wei Li, Chunlei Guo, Mohamed ElKabbash

**Affiliations:** GPL Photonics Lab, State Key Laboratory of Applied Optics, Changchun Institute of Optics, Fine Mechanics and Physics, Chinese Academy of Sciences, Changchun 130033, China; University of Chinese Academy of Science, Beijing 100039, China; The Institute of Optics, University of Rochester, Rochester, NY 14627, USA

**Keywords:** angularly selective system, radiative cooling, thermal design, thin film design

## Abstract

We theoretically analyze the impact of angular selectivity on the radiative cooling performance of thermal emitters. We investigate the effect of spectral selectivity, environmental conditions, and parasitic heating on the minimum possible equilibrium temperature of the thermal emitter. We show that combining angular and spectral selectivity is necessary to reach deep subfreezing temperatures. We also show that angularly selective thermal emitters increase the cooling performance in humid environments, however, they require management of nonradiative heat transfer processes. We introduce a general scheme to realize angularly and spectrally selective absorption/emission using a thin film stack consisting of an angle dependent transmission filter overlayed on a selective thermal emitter. The thermal emitter total thickness is ∼16 μm, an order of magnitude less than previously proposed angular selective thermal emitters/absorbers and operates under s- and p-polarized light without using anisotropic layers. Under realistic conditions and reasonable parasitic heating, the proposed emitter can be cooled down to Δ*T* = −46 °C below ambient temperature. Our work highlights the advantages and drawbacks of angular selective thermal emitters towards practical and efficient radiative cooling devices.

## 1 Introduction

Approximately 70% of incident solar radiation is absorbed by the Earth. The Earth cools itself down by radiating infrared wavelengths to the cold outer space (3 K) which acts as Earth’s heat sink. Most of the radiated wavelengths are absorbed by the atmosphere except within the atmospheric transparency window (8–13 μm). Accordingly, an object on earth emitting at these wavelengths *sees* the outer space and heat transfer occurs between the two objects, cooling the Earthian object. Given that radiative cooling requires no energy consumption, it is crucial to exploit its potential to minimize our reliance on energy-intensive compression-based air conditioners [1, 2]. Air conditioning and refrigeration consumes 15% of the total electricity generated in the world, mainly through fossil fuels. Radiative cooling can significantly reduce the carbon footprint of the cooling sector and be a major thrust towards a global net zero carbon emission.

The quantity that determines an object’s ability to thermally radiate at a given wavelength is the emissivity *ɛ*(*λ*, *θ*). According to Kirchhoff’s law of thermal radiation, an object’s emissivity $\varepsilon \left(\lambda ,\theta \right)$ is equal to its absorptivity $\alpha \left(\lambda ,\theta \right)$ at a given wavelength and angle. This explains the observation that a black radiator facing a clear night sky can reach sub-ambient temperature [3, 4]. In ancient Persia and India, nocturnal ice making was possible in deserts at night thanks to radiative cooling. Modern efforts attempted to realize subambient daytime and nighttime radiative cooling to replace or reduce the reliance on compressive air conditioning, to reach deep low temperatures passively to cool a given object to subfreezing temperatures [3, 5, 6], and to generate energy at night through thermoelectric generation [7–10].

Subfreezing temperatures were realized through employing a spectrally selective thermal emitter and reducing parasitic (convective and conductive) heating and solar irradiance [3]. Spectral selectivity refers to engineering the thermal emission of the thermal emitter to have high emissivity/absorption only at wavelengths where the atmosphere is transparent (weakly emissive), while having near zero emissivity/absorption at wavelengths where the atmosphere is opaque (emissive).

Directional (angularly selective) thermal emission was considered to increase the net radiated power by blocking solar irradiance [3, 11], block thermal emission from surrounding opaque and highly emissive buildings [12] or to maximize the radiated power from an omnidirectional thermal emitter [13]. Angular selectivity, however, can play a similar role to spectral selectivity in minimizing the absorbed radiation from the atmosphere [14]. At higher angles, measured from the zenith, atmosphere’s emissivity increases as it becomes thicker. Gentle and Smith considered angular selectivity of thermal emission in the context of experimental data on directionally emitting coatings [15] and suggested that angular selective thermal emission can enhance the net radiated power from thermal emitters. A more recent work theoretically showed that angular selectivity can significantly improve the cooling performance of thermal emitters compared to isotropic thermal emitters and generalized their findings to slanted emitters.

However, the advantages of angular selectivity come with a cost-while limiting the selectivity increases the temperature range with a net positive difference between radiated and absorbed thermal radiation, it reduces the total radiated heat flux $Q_{\text{net}}^{\text{Rad}}$ as it limits the wavelength and angular range where radiation takes place. Here, we investigate how angular selective thermal emission can improve radiative cooling under various conditions by considering the effect of angular and spectral selectivity on the cooling performance in (i) ideal conditions, (ii) in environments with high humidity and low atmospheric transparency, and (iii) when parasitic heating is present. We conclude that angular selectivity is only advantageous if employed alongside with spectral selectivity that it improves the performance of radiative cooling in environments with low atmospheric transparency, e.g., humid regions, and that high parasitic heating conditions favor isotropic thermal emitters. We introduce a realistic angularly and spectrally selective thermal emitter by combining a highly iridescent transmission filter on a Si_3_N_4_ thermal emitter. The thermal emitter total thickness is 16 μm and consists of 10 layers which is orders of magnitude thinner and less in terms of number of layers than previously proposed angularly selective thin films [16–18]. The proposed emitter and can cool down an object to 60 °C below ambient temperature under ideal conditions and to 40 °C below ambient temperatures under experimentally realizable conditions. Our results highlight the advantages and drawbacks of angular selective thermal emission under various conditions and introduce a general approach to realize lithographically free and subwavelength thick angularly selective thermal emitters.

## 2 Results and discussions

### 2.1 Theoretical background

**Figure 1:**
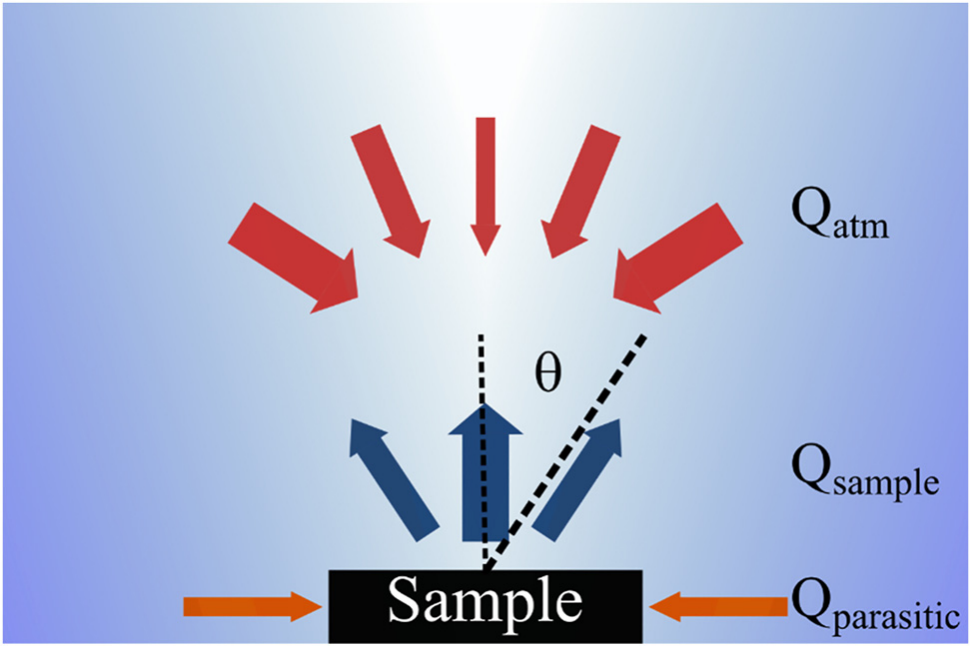
Geometry of the problem: we consider the thermal emission of a sample that can be either spectrally selective or a blackbody. The heat flux radiated by the sample *Q*_sample_, cools it down. The sample absorbs thermal radiation from the atmosphere *Q*_atm_. The thermal emissivity of the atmosphere *ɛ*_atm_ depends on the angle from the sample’s normal. The sample is subjected to parasitic heating from its environment *Q*_parasitic_. The net radiation determines the equilibrium temperature of the sample. Our goal is to design the angular absorption/emission of the sample to minimize absorption of thermal radiation from the atmosphere by introducing angular selective absorption/emission.

We analyze the performance of a thermal emitter that is directly facing the sky with no side shades. The net heat flux from a thermally emitting sample *Q*_net_ (see Figure 1) is given by,

(1) $$Q_{\text{net}}\left(T\right)=Q_{\text{sample}}\left(T\right)-Q_{\text{atm}}\left(T_{\text{atm}}\right)-Q_{\text{sun}}-Q_{\text{parasitic}}$$

*Q*_sample_ is the emitted heat flux from the thermal emitter and is given by:

(2) $$Q_{\text{sample}}\left(T\right)=\int d\Omega \cos\theta \int_{0}^{\infty }d\lambda I_{BB}\left(T,\lambda \right)\varepsilon \left(\lambda ,\theta \right)$$

where $\int d\Omega =2\pi \int_{0}^{\pi /2}d\theta \sin\theta$ is an integral over the hemispherical solid angle and $I_{BB}\left(T,\lambda \right)=\frac{2hc^{2}}{\lambda^{5}}\frac{1}{e^{hc/\lambda k_{B}T}-1}$ is the intensity of the blackbody radiation at a given temperature *T* of the thermal emitter, *h* is plank’s constant, *c* is the speed of light, *k*_B_ is the Boltzmann constant, and *λ* is the wavelength. $\varepsilon \left(\lambda ,\theta \right)$ is the angularly and spectrally dependent emissivity of the sample. *Q*_atm_ is the thermal radiated heat flux of the atmosphere that is absorbed by the sample and is given by:

(3) $$\begin{array}{cc} Q_{\text{atm}}\left(T_{\text{atm}}\right) & =\int d\Omega \cos\theta \int_{0}^{\infty }d\lambda I_{BB}\left(T_{\text{atm}},\lambda \right)\\ & \;\times \varepsilon \left(\lambda ,\theta \right)\varepsilon_{\text{atm}}\left(\lambda ,\theta \right) \end{array}$$

$I_{BB}\left(T_{\text{atm}},\lambda \right)$ is the intensity of the blackbody radiation of the atmosphere at ambient temperature*T*_atm_, here taken as 293 K. *ɛ*_atm_(*λ*, *θ*) is the spectrally and angularly dependent thermal emissivity of the atmosphere. For most of the results below, $\varepsilon_{\text{atm}}\left(\lambda ,\theta \right)=1-{\tau_{\text{atm}}}^{1/\cos\left(\theta \right)}$ , where *θ* is the polar angle, and *τ*_Atm_ is the atmospheric transmittance [3]. The atmospheric emissivity is assumed to have no dependence on the azimuthal angle. Moreover, *τ*_atm_ = 0.8 is assumed within the IR atmospheric transmission window (8–13 μm) and zero everywhere else for simplicity. We define $Q_{\text{net}}^{\text{Rad}}=Q_{\text{sample}}-Q_{\text{atm}}$ as the net radiative heat flux. *Q*_sun_ is the heat flux absorbed by the sample due to solar irradiation and is assumed to be zero here as we consider only selective thermal emitters that have negligible absorption at wavelengths corresponding to the solar spectrum. *Q*_parasitic_ is the heat flux absorbed by the sample due to convection and conduction from its interaction with the environment and is given by:

(4) $$Q_{\text{parasitic}}=h\left(T_{\text{atm}}-T\right)$$

where *h* is an effective heat transfer coefficient. Our goal is to minimize the equilibrium temperature of the sample *T*. The equilibrium temperature is the temperature that satisfies the condition *Q*_net_ = 0. Since *Q*_sample_ decreases for lower *T*, i.e., lower *I*_BB_, while the atmosphere is at a fixed temperature, the desired thermal emitter is one that maximizes the difference between *Q*_sample_ and *Q*_atm_ to obtain the lowest possible temperature. Spectrally selective thermal emitters are engineered to $\varepsilon \left(\lambda ,\theta \right)∼0$ at wavelengths where *ɛ*_atm_(*λ*, *θ*) ∼ 1, i.e., away from the atmospheric window, and to have near unity $\varepsilon \left(\lambda ,\theta \right)∼1$ at wavelengths within the atmospheric transmission window. However, at higher angles, *ɛ*_atm_(*λ*, *θ*) approaches unity even within the atmospheric window. This means that *Q*_Atm_ is high at all wavelengths for larger angles. An angularly selective thermal emitter can overcome this problem by having near zero *ɛ*(*λ*, *θ*) at higher angles where $\varepsilon_{\text{atm}}\left(\lambda ,\theta \right)$ is high.

To calculate the effect of angular selectivity, we assume that *ɛ*(*λ*, *θ*) = 1 up to a certain cutoff polar angle *θ*_Cuttoff_ beyond which that *ɛ*(*λ*, *θ*) = 0. In the following, we model different conditions where we examine the effect of angular selectivity on *T*.

### 2.2 Effect of angular selectivity on spectrally selective versus blackbody thermal emitters

**Figure 2:**
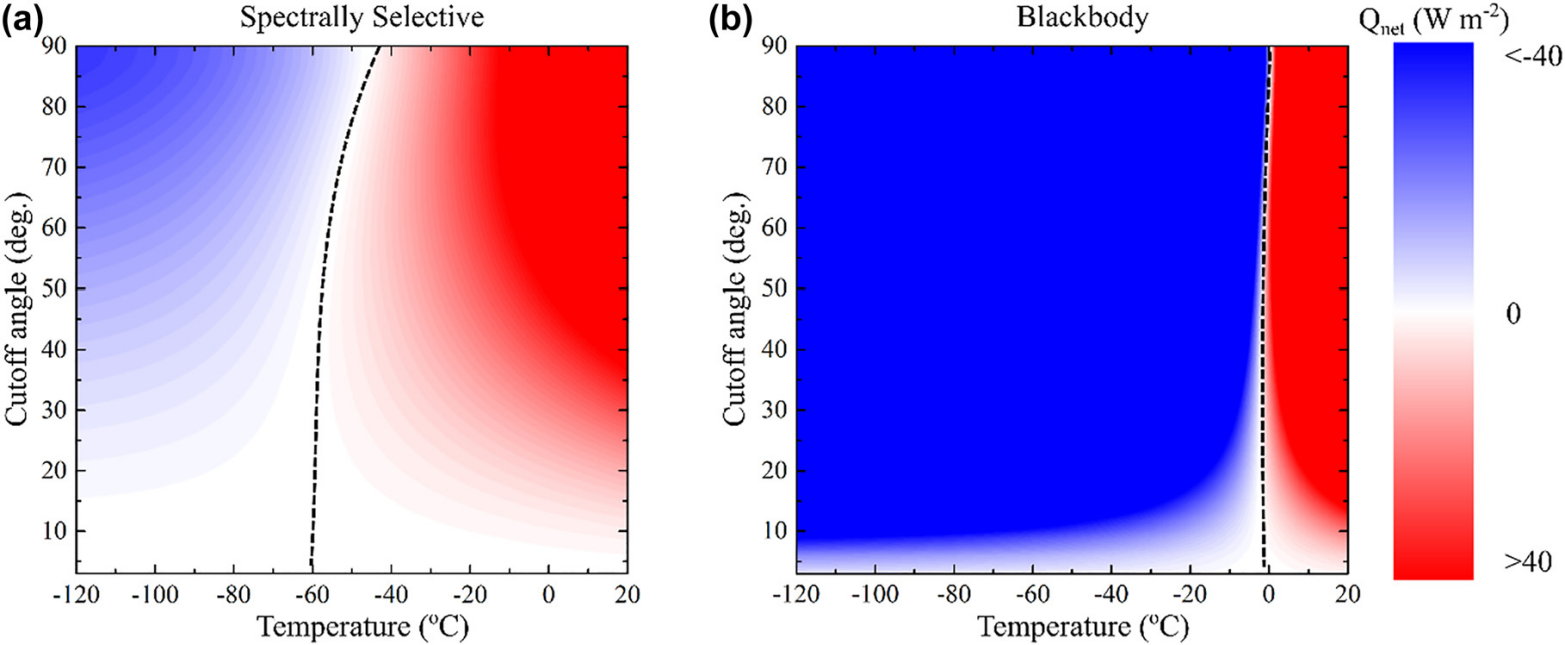
Effect of angular selectivity on the steady state temperature of (a) a spectrally selective thermal emitter, and (b) a blackbody emitter. The dashed line traces the values of *Q*_net_ = 0 where the corresponding temperature is the equilibrium temperature of the emitter. Angular selectivity plays little role in minimizing the blackbody emitter temperature.

We calculate *Q*_net_ as a function of the sample temperature and the cutoff angle for spectrally selective thermal emitter (Figure 2a) versus a blackbody emitter (Figure 2b). Here we assume *Q*_parasitic_ = 0. The dashed line traces the *Q*_net_ = 0 condition corresponding to the equilibrium sample temperature. For a *θ*_Cuttoff_ = 90°, i.e., the sample is not angularly selectivity, the steady state temperature is ∼−46 °C and ∼0 °C for the spectrally selective and blackbody emitters, respectively, in agreement with other works [3]. For high angular selectivity, e.g., *θ*_Cuttoff_ ∼ 1°, the steady state temperature is ∼−64 °C and −2 °C for the spectrally selective and blackbody emitter, respectively. Angular selectivity, hence, is only advantageous for spectrally selective emitters. For a blackbody emitter, angular selectivity plays a minor role in changing the net heat flux emitted by the blackbody. Note that below *θ*_Cuttoff_ ∼ 40°, the selective emitter experiences little change in the steady state temperature.

### 2.3 Effect of humidity on angularly selective thermal emitters

The atmospheric emissivity depends strongly on the geographic location and climate conditions. The level of water content in the atmosphere also greatly influences atmospheric emissivity in the atmospheric window. For example, using a similar setup as Raman et al. [2] who obtained 40.1 W/m^2^ daytime radiative cooling power at California, Tso et al. [19] conducted a field test in Hong Kong and showed that radiative sky cooling does not perform as well under a humid and cloudy climate. The researchers found that the maximum nighttime cooling power was only 38 W/m^2^ with a clear sky and did not observe any daytime cooling effect.

**Figure 3:**
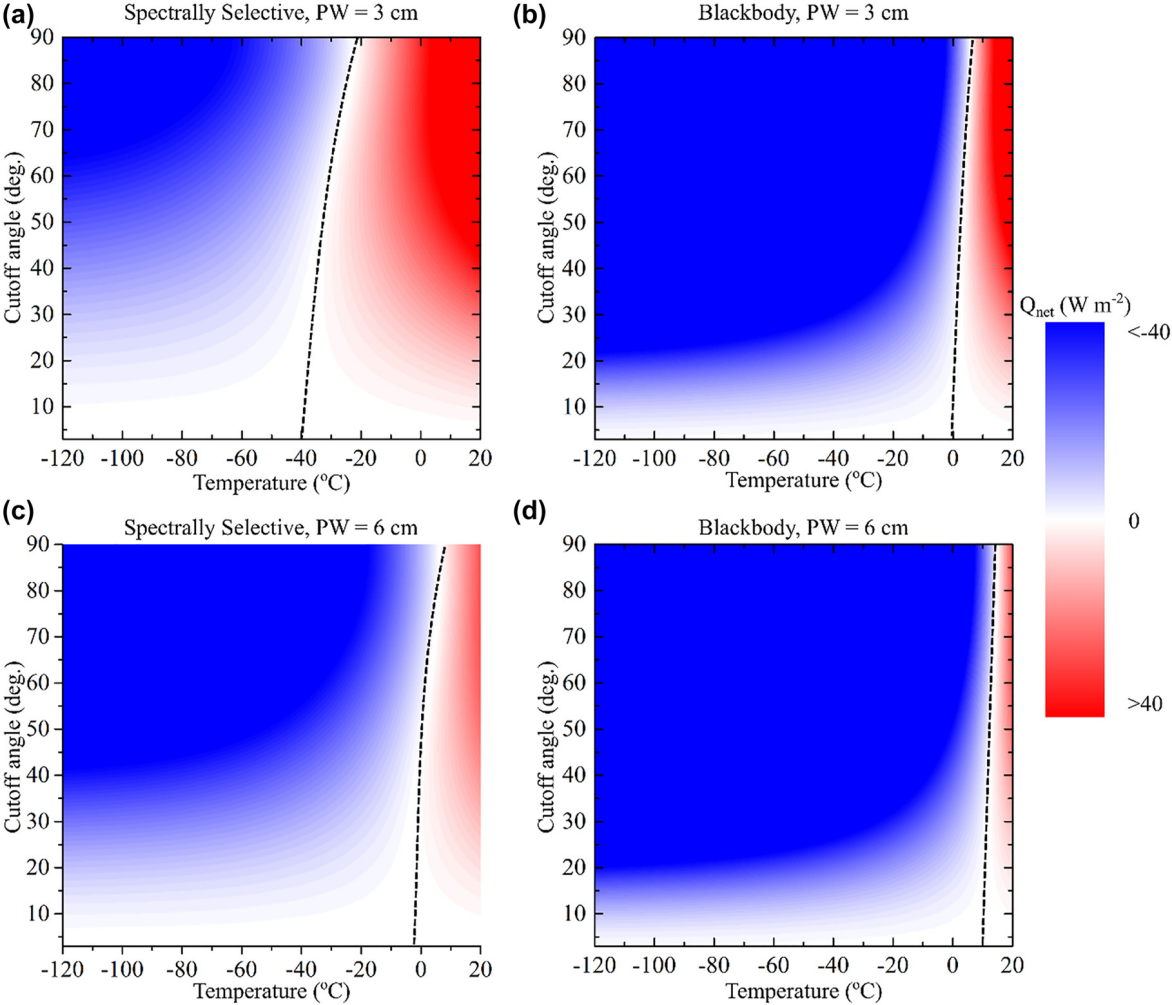
Effect of humidity on the performance of angularly selective thermal emitter. For spectrally selective emitter, subfreezing temperatures are attainable for all angles at (a) 3 cm precipitable water (PW) level for all angles, and (c) for a cutoff angle <30° for a PW level of 6 cm. For a blackbody, angular selectivity can improve the emitter performance slightly but subfreezing temperatures are not possible at any cutoff angle.

We investigate the effect of angular selectivity on the steady state temperature of a spectrally selective emitter and a blackbody emitter. The level of water content in the atmosphere is quantified in precipitable water units, i.e., the depth of water in a column assuming that all the water in that column is precipitated as rain. We calculate the angular dependence of the steady state temperature of a thermal emitter assuming an ambient temperature of ∼20 °C under two precipitable water (PW) levels of 3 cm and 6 cm which corresponds to an average atmospheric transmittance *τ*_Atm_ ∼ 0.7 and 0.35, respectively [20]. We again assume *Q*_Parasitic_ = 0. A spectrally selective emitter reaches sub-freezing temperature of ∼−20 °C for *θ*_Cuttoff_ = 90° (Figure 3a). Introducing angular selectivity can reduce the steady state temperature down to ∼−40 °C (Figure 3a). If the precipitable water level increases to 6 cm, a spectrally selective emitter steady state temperature is ∼6 °C for *θ*_Cuttoff_ = 90°, and while an angularly selective emitter temperature can drop down to ∼−3 °C for a cutoff angle below 30° (Figure 3b). On the other hand, the steady state temperature of a blackbody does not benefit from angular selectivity and remains above the freezing temperature for all angles (Figure 3c and d). These results are significant. Angular selectivity can lower the steady state temperature to subfreezing temperatures even for environments with high levels of humidity assuming parasitic losses are eliminated.

### 2.4 Effect of parasitic losses on angularly selective thermal emitters

Parasitic Heat losses in the form of convective and conductive heat transfer can degrade the performance of passive radiative coolers significantly. Introducing selectivity, spectral, and angular, necessarily means reducing *Q*_sample_. The reason why selectivity can reduce the emitter’s equilibrium temperature is because it marginally decreases *Q*_atm_ such that a positive $Q_{\text{net}}^{\text{Rad}}$ is obtained at lower temperatures ignoring. On the other hand, *Q*_parasitic_ does not depend on the optical properties of the sample or the atmosphere and cannot be optimized by engineering the optical properties of the thermal emitters leading to lower *Q*_net_.

**Figure 4:**
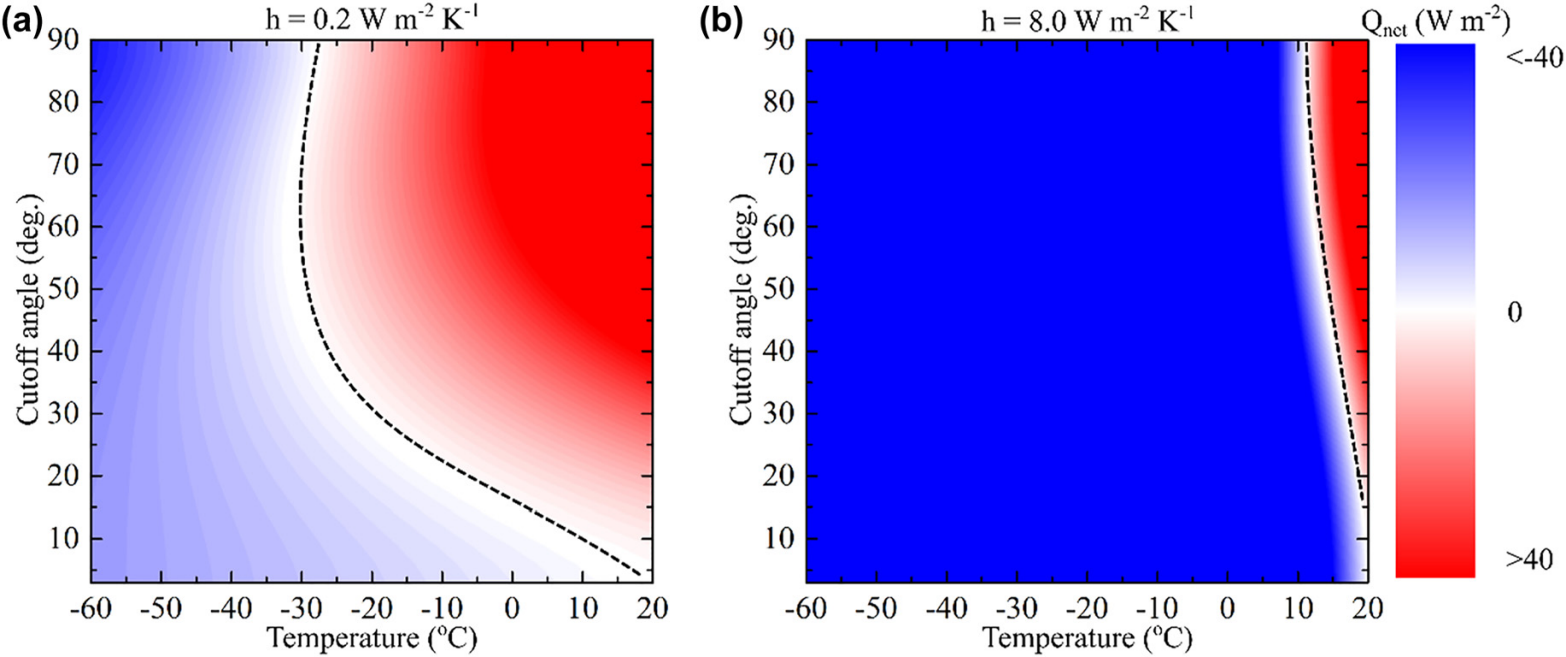
Effect of parasitic heating on the performance of angularly and spectrally selective thermal emitter at an effective heat transfer coefficient (a) *h* = 0.2 W m^−2^ K^−1^ and (b) *h* = 8 W m^−2^ K^−1^. Decreasing the cutoff angle does not guarantee a better performance due to the decrease in *Q*_sample_. We find an optimal *θ*_Cuttoff_ ∼55° for (a), while for (b) decreasing the cutoff angle increases the equilibrium temperature.

Figure 4 shows the effect of introducing *Q*_parasitic_ for *h* = 0.2 W m^−2^ K^−1^ (Figure 4a) and *h* = 8 W m^−2^ K^−1^ (Figure 4b). For *h* = 0.2 W m^−2^ K^−1^, we find an optimal *θ*_Cuttoff_ ∼55° that leads to a steady state temperature of −33 °C, i.e., 5 °C lower than *θ*_Cuttoff_ = 90°. On the other hand, for *h* = 8 W m^−2^ K^−1^ angular selectivity only increases the equilibrium temperature. Consequently, angular selectivity is a promising approach only if parasitic losses are well managed. Note that Jeon and Shin [21] concluded that directional thermal emitters tolerate parasitic losses which, according to our results, is only true for low parasitic heating values where a thermal emitters is placed in a low pressure chamber at 10^−5^–10^−6^ Torr [3].

## 3 Realization of thin film based angularly selective thermal emitter

**Figure 5:**
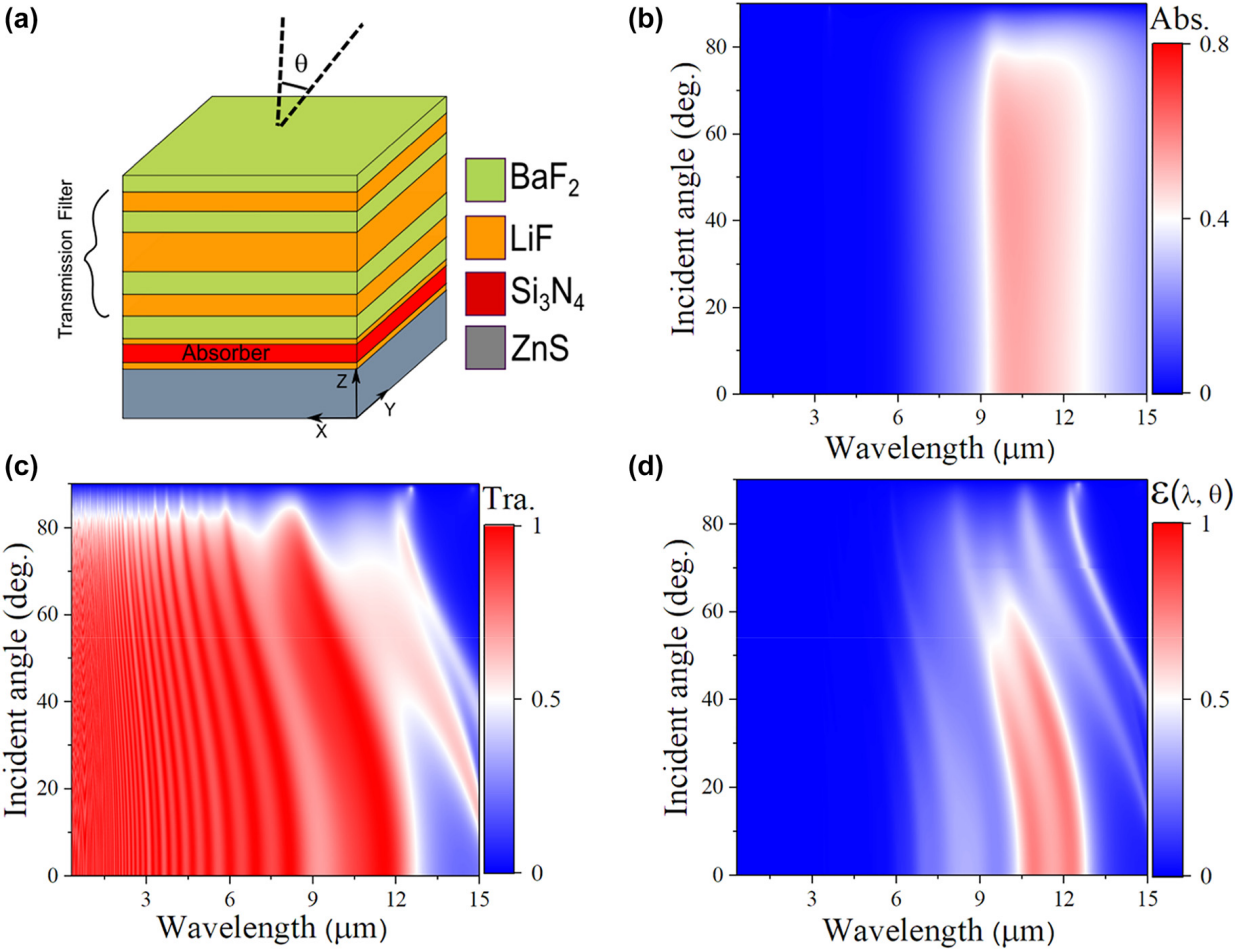
A practical spectrally and angularly selective thermal emitter: (a) Schematic of proposed emitter consisting of a transmission filter on top of a thermal emitter. (b) Average s- and p-polarized) angular absorptance for 0.8 μm thick Si_3_N_4_. (c) Average s- and p-polarized) angular transmission of a transmission filter consisting of BaF_2_ – LiF – BaF_2_ – LiF – BaF_2_ – LiF – BaF_2._ (d) (Average s- and p-polarized) angular emissivity for the thermal emitter, shown in (a).

We discuss the realization of a spectrally and angularly selective thermal emitter. While directional thermal emission is certainly possible using metamaterials and metasurfaces [22–25], thin-film based thermal emitters/absorbers are more practical as these designs can be scaled with low fabrication costs and are more durable [26–30]. FDTD Ansys Lumerical^®^ is used for all optical simulations for all optimizations using transfer matrix method and FDTD simulation window (see Supplementary Figure 1). Then, with the absorption response obtained from an optimized stack, we model Eq. (1) using FDTD scripting. Our design includes two elements: a spectrally selective thermal emitter and a transmission filter with high angular dependence [31] (Figure 5a). Si_3_N_4_ enjoys strong absorption in the atmospheric window associated with its phonon–polariton resonance [32, 33] (see Supplementary Figure 2) The mirror consists of two dielectric films (Barium fluoride BaF_2_ and lithium fluoride LiF) that enjoy high index contrast such that they act effectively as a dielectric mirror [34] (see Supplementary Figure 2). The calculated average (s- and p-polarized) absorption of the absorber layer is shown in Figure 5b.

The transmission filter is a semitransparent Fabry–Perot cavity consisting of a low index dielectric sandwiched between two CsF and LiF dielectric mirrors. We choose LiF [34] as our dielectric due to its low index (∼1) in the IR range which leads to highly iridescent resonances [29–31] (see Supplementary Figure 2(a)). The effect of LiF layer thickness is discussed (see Supplementary Figure 5). The angular transmission of the transmission filter is shown in Figure 5c. Clearly, the transmission within the absorption band of the Si_3_N_4_ (Figure 5b) decreases significantly for angles >50°. By overlaying the transmission filter on the thermal emitter, we obtain an angularly selective thermal emitter with unity emissivity for angles below 50° and significantly low emissivity for angles >50° as shown in Figure 5d (see Supplementary Figure 6). Note that the absorption/emission ∼0 within the solar spectrum which validates the assumption that *Q*_sun_ = 0. The entire stack is placed on a zinc selenide (ZnS) substrate due to its high mechanical stability, high transmission in the atmospheric window, and negligible absorption in solar window [35, 36] (see Supplementary Figure 4). The optimized top-to-bottom thicknesses of each layer in the stack are as follows: BaF_2_: 1.8 μm, LiF: 2.9 μm, BaF_2_: 0.1, LiF: 3 μm, BaF_2_: 1.8 μm, LiF: 2.9 μm, BaF_2_: 1.8 μm, LiF: 0.5 μm, Si_3_N_4_: 0.8 μm, LiF: 0.5 μm. Adding 0.5 μm LiF films above and below the Si_3_N_4_ layer minimizes the high absorption of Si_3_N_4_ at high angles for p-polarized light by ensuring that the Brewster angle for the top and bottom interfaces of Si_3_N_4_ are significantly different.

The emitter has a thickness of 16 μm, which is more than 2 orders of magnitude thinner and 3 orders of magnitude less in terms of number of layers than previously proposed angularly selective thin films [16–18]. Angular selective thermal emission was theoretically proposed [17] using a stack of various 1D photonic crystals that reflect a broad wavelength range except at their Brewster angle where light is transmitted. This approach requires a stack of ∼10^3^ layers with a total thickness of 10^4^ nm, and thus is not practical. In addition, it offers limited control over the angular and spectral range where the angular and spectral selectivity take place. On the other hand, our approach requires a total thickness of ∼16 μm and 10 layers. It also provides broad angular and spectral band control, as well as tunability because of the dielectric spacer layer in the transmission filter. In addition, our proposed thermal emitter shows excellent tolerance to fabrication errors (see Supplementary Figures 7 and 8).

**Figure 6:**
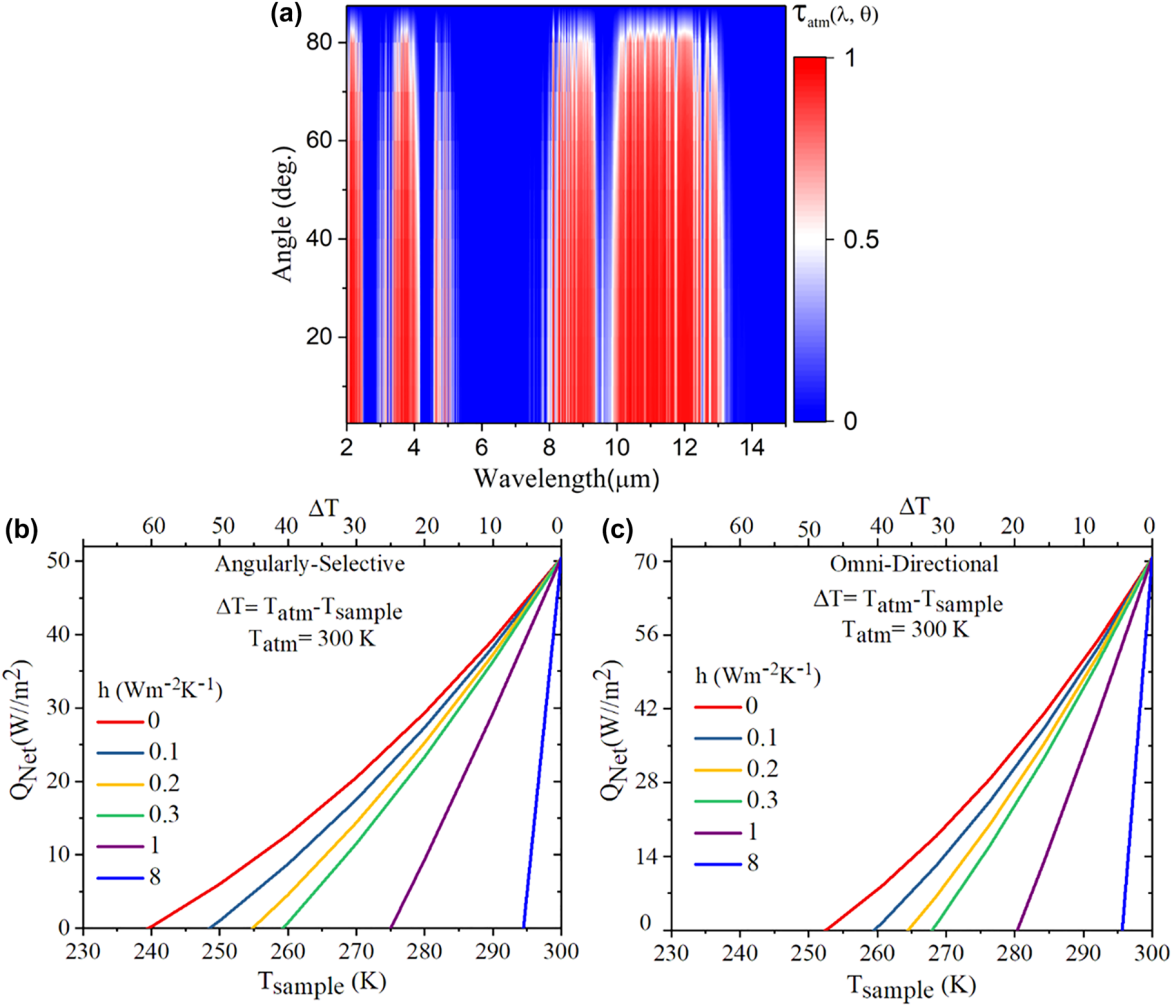
Steady state temperature of thermal emitter. (a) Atmospheric emissivity as a function of angle and wavelength at relative humidity of 20%. Net power out ***Q***_**net**_ as a function of the sample temperature ***T***_**sample**_ at different parasitic heat transfer coefficient heat *h* keeping atmospheric temperature ***T***_**atm**_ at 300 K for (b) the porposed angularly and spectrally selective thermal emitter and (c) the same thermal emitter without the iridescent transmission filter shown in Figure 5a. The steady state temperature and temperature difference (Δ*T* = *T*_atm_ − *T*_sample_) corresponding to *Q*_net_ = 0 for different *h*(W m^−2^ K^−1^) = 0, 0.1, 0.2, 0.3, 1 and 8 are 60 °C, 51 °C, 45 °C, 40 °C, 25 °C and 6 °C, respectively for (b) the proposed angularly and spectrally selective emitter and 48 °C, 40 °C, 35 °C, 31 °C, 19 °C and 4 °C for (c) the spectrally selective emitter.

To evaluate the performance of our proposed angularly and spectrally selective thermal emitter, we calculate its equilibrium temperature as a function of parasitic losses. We consider atmospheric transmission *τ*_atm_(*λ*, *θ*) as a function of angle and wavelength (Figure 6a). We obtained the spectral angular transmittance *τ*_atm_(*λ*, *θ*) of the atmosphere using standard commercial software (ModTran5) [37], at different wavelengths and incident angles. As the transparency of the atmosphere strongly depends on the amount of water vapor, we calculated the *τ*_atm_(*λ*, *θ*) for relative humidity of 20% as shown in Figure 6(a). The spectral angular emittance of the atmosphere is *ɛ*_atm_(*λ*, *θ*) = 1 − *τ*_atm_(*λ*, *θ*). Figure 6(b) shows calculated steady state temperature (*T*_steady state_ = *T*_atm_ − *T*_sample_) and net power output using Eq. (1) for various *h* values. The sample’s minimum attainable temperature is *T*_steady state_. For *h* = 0 and 8 W m^−2^ K^−1^ *T*_steady state_ are 60 °C and 5 °C, respectively. Finally, in Figure 6(c) we calculate the steady state temperature of omnidirectional emitter without the transmission filter at different *h* values. The results show that significant performance drop for the spectrally selective thermal emitter without the iridescent transmission filter that enables angular selectivity. However, the performance advantage of angular selectivity is only significant for low parasitic losses. Accordingly, angularly selective thermal emitters are valuable for radiative cooling, provided that the parasitic losses in the system are minimized.

## 4 Conclusion and outlook

Ultimately, the usefulness of introducing angular selective thermal emission depends on its performance under realistic conditions and on the ability to realize scalable and inexpensive angularly selective emitters. To this end, we studied the performance of angularly selective thermal emitters under various conditions by investigating the effect of spectral selectivity, environmental conditions, and parasitic heating. We concluded that angularly selective thermal emitters perform better when considering to environmental effects which significantly widens the scope of application and relevance of radiative cooling schemes. However, angular selectivity is decreasing the total radiated power and thus parasitic heating is detrimental to its performance. Hence, angular selectivity requires management of non-radiative heat transfer processes. The use of vacuum chambers at high vacuum conditions of 10^−5^ Torr is necessary unless other solutions are proposed. Such low pressures require Turbo pumps which are not practical. For example, the heat conduction of air can be reduced if the chamber is initially filled with a monoatomic gas. Studying the heat transfer at pressures where mechanical pumps are used (>10^−3^ Torr) is also necessary. In that case, air’s heat conductivity can be simply reduced by placing the sample further away from the chamber walls. A future work should investigate angular selective thermal emission from samples surrounded by a conical high reflectance mirror which can eliminate the tradeoff between *Q*_sample_ and $Q_{\text{net}}^{\text{Rad}}$ . We also introduced a general scheme to realize angular selective absorption/emission using thin film stack consisting of an angle dependent transmission filter overlayed on a selective thermal emitter which provided the desired angle dependent thermal emission. The proposed thin film stack is an order of magnitude thinner than previously proposed thin-film based angularly selective absorbers and can be extended to other wavelength ranges.

## Supplementary Material

Supplementary Material Details
