# Supplementary material for: Angularly selective thermal emitters for deep subfreezing daytime radiative cooling
Source: Nanophotonics. 2022 Aug 5;11(16):3709–17. doi: 10.1515/nanoph-2022-0032 (PMC11501671; doi:10.1515/nanoph-2022-0032)
Supplement: Supplementary file 1 — Supplementary Material Details [file j_nanoph-2022-0032_suppl.docx]

**Supplementary Information**

Angularly selective thermal emitters for deep subfreezing daytime radiative cooling

Sandeep Kumar Chamoli^1,2^, Wei Li^1,2*^, Chunlei Guo^3*^ and Mohamed ElKabbash^3,4*^

*^1^ GPL, State Key Laboratory of Applied Optics, Changchun Institute of Optics, Fine Mechanics and Physics, Chinese Academy of Sciences, Changchun 130033, China*

*^2^ University of Chinese Academy of Science, Beijing 100039, China*

*^3^ The Institute of Optics, University of Rochester, Rochester, New York 14627, USA.*

*^4^ Current address: The Research Laboratory of Electronics, Massachusetts Institute of Technology, Cambridge, Massachusetts 02139, USA*

**Corresponding author email:* [weili1@ciomp.ac.cn](mailto:weili1@ciomp.ac.cn); [guo@optics.rochester.edu](mailto:guo@optics.rochester.edu); [melkabba@mit.edu](mailto:melkabba@mit.edu)

**Simulation method:**

For the simulations, we used both the Transfer Matrix Method (TMM) and Finite Difference Time Domain Simulation (FDTD) based on Ansys Lumerical^®^ to ensure reliability and accuracy. As TMM is quite fast and requires less simulation time, it is used to study the optical response of structures with different thicknesses, and layers. Supplementary Figure 1 illustrates a simulation region with a plane TE-polarized wave source with a wide spectral range (0.3 μm - 15 μm) and angular range (0^O^ - 90^O^). However, all the calculations are done by taking average of TE and TM polarised light. Light incidents from the top of the device in the negative Z direction, reflection and transmission are collected from the reflection and transmission monitors on the top and bottom sides of the emitter. The absorption is the difference between reflection and transmission, measured at the reflection and transmission monitors, respectively. Perfectly matched layers (PML) are used in the light propagation direction (Z) and periodic boundary conditions are used in the X direction.


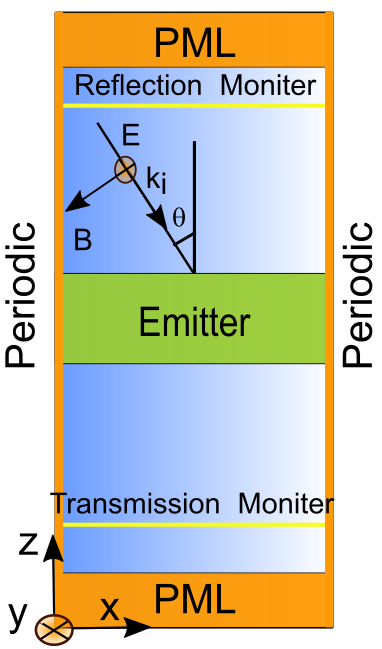


**Supplementary Figure 1.** Schematic of simulation geometry

The emitter is composed of the following 10 layers: BaF_2_ (Barium fluoride) - LiF (Lithium fluoride) – BaF_2_ - LiF - BaF_2_ - LiF- BaF_2_- LiF - BaF_2_ -LiF- Si_3_N_4_ (Silicon nitride)-LiF- ZnS (Zinc sulfide). CsF - LiF work effectively as a dielectric mirror (DM). A refractive index of BaF_2_ and LiF has been calculated using following dispersion and is shown in figure 2 (a) ^1^.

BaF_2_:

$$n^{2}=0.33973+\frac{.81070\lambda^{2}}{\lambda^{2}-{0.10065}^{2}}+\frac{.19602\lambda^{2}}{\lambda^{2}-{29.87}^{2}}+\frac{4.52469\lambda^{2}}{\lambda^{2}-{53.82}^{2}}+1$$

LiF:

$$n^{2}=\frac{.92549\lambda^{2}}{\lambda^{2}-{0.07376}^{2}}+\frac{6.96747\lambda^{2}}{\lambda^{2}-{32.792}^{2}}+1$$

Since Si_3_N_4_ shows high absorption in the atmospheric transmission window and no absorption within the solar window (see figure 2(b)), it is one of the most widely used materials for radiative cooling ^2–4^. Middle LiF layer forms a cavity between the two DM and has a very low refractive index in the atmospheric window, which makes our transmission filter iridescent, i.e., angle dependent^5^.


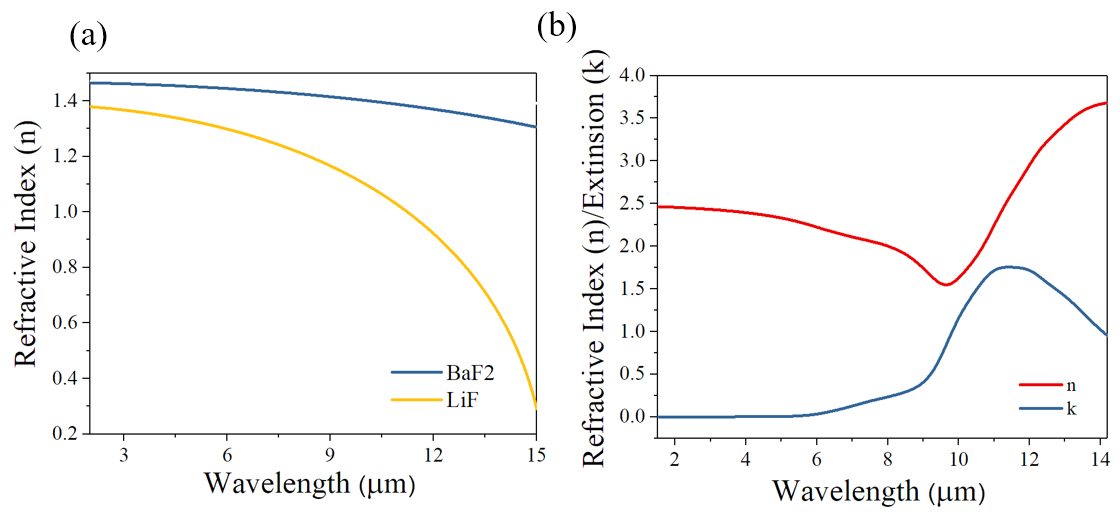


**Supplementary Figure 2.** Refractive index of fluoride materials, (b) refractive index (n) and extinction coefficient values (k) of Si_3_N_4_.

The ZnS substrate is an excellent choice due to its high mechanical stability, transparent in the atmospheric window and not susceptible to damage from solar ultraviolet ^6^. The index of ZnS is determined using the following dispersion in figure 4 ^7^:

$$n^{2}=\frac{.14388\lambda^{2}}{\lambda^{2}-{0.2421}^{2}}+\frac{4430.99\lambda^{2}}{\lambda^{2}-{36.71}^{2}}+8.393$$




**Supplementary Figure 3.** Refractive index and extinction coefficient of ZnS

Supplementary Figure 4 illustrates the effect of the LiF cavity layer thickness on transmission filter (BaF_2_-LiF- BaF_2_- LiF-BaF_2_-LiF- BaF_2_) resonance with different incident angles of $\theta=0^{o}, {30}^{o}, {60}^{o}$ and ${80}^{o}$, in figure 4 (a), (b), (c) and (d), respectively. Generally, the transmission filter resonance is the result of constructive interference ^8^:

($m+\frac{1}{2})\lambda_{m}=2d_{LiF}n(\lambda)$

Where $d_{LiF}$ is the layer thickness, and $n\left( \lambda\right)$ is the wavelength dependent refractive index. Its clear resonance varies with the layer thickness and incident angle $\theta$. It is clear that the resonance is varying as a function of LiF thickness and incident angle. The resonance begins to appear in the wavelength range of 10-12 μm from normal incidence. As the incident angle increases, the resonance becomes narrower and more reflecting. By choosing $d_{LiF}$ = 3 μm, the filter will be angularly selective for angles less than 60^o^ in the wavelength range of interest.


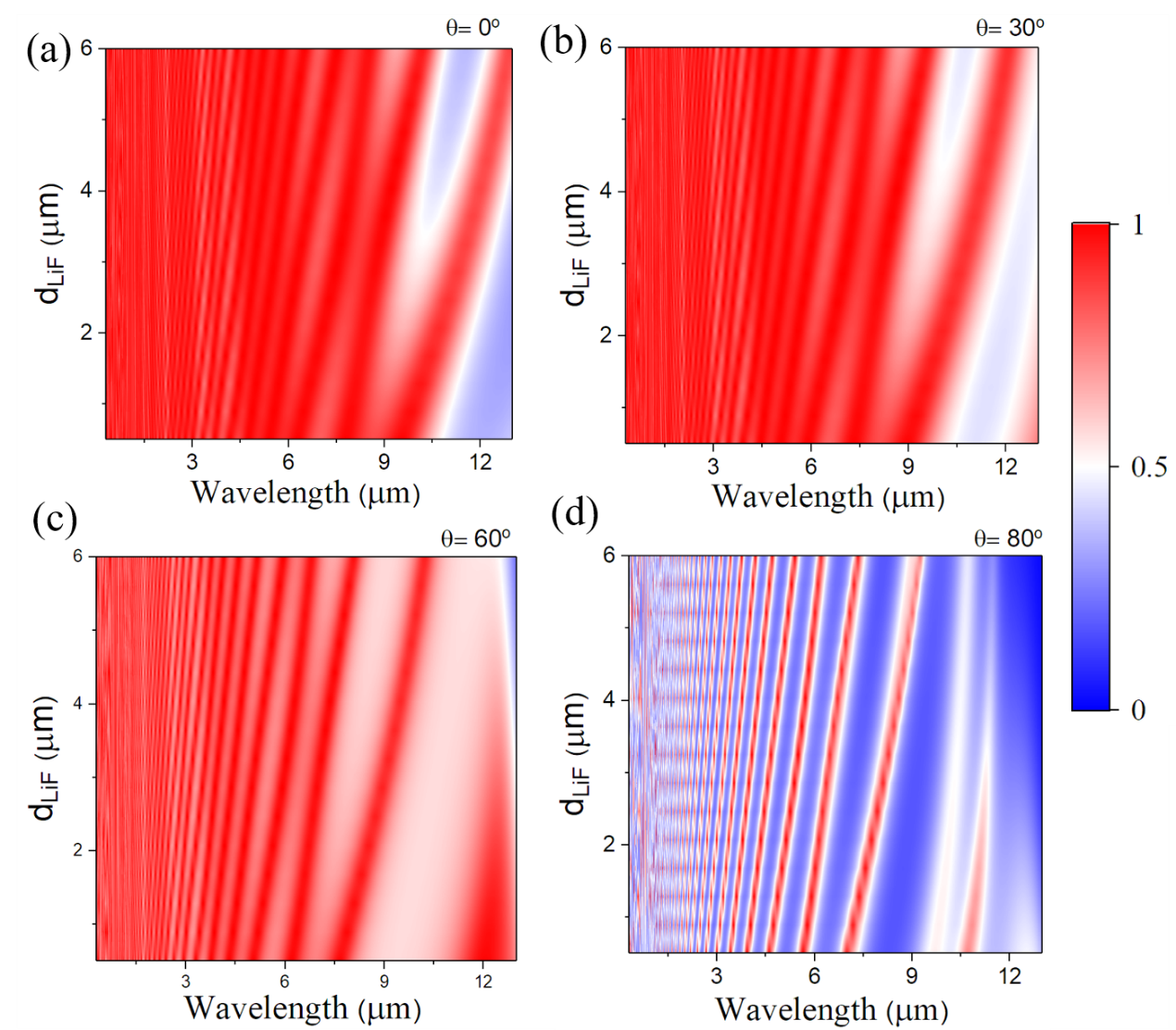


**Supplementary Figure 4.** Average transmission as a function of incident wavelength and LiF cavity layer thickness for (a) θ=0^o^, (b) θ=30^o^, (c) θ=60^o^ and (d) θ=80^o^, for the transmission filter: (BaF_2_ (1.8 μm)-LiF(2.9 μm)- BaF_2_ (0.1 μm) - LiF (d_LiF_) - BaF_2_ (1.8 μm)-LiF(1.8 μm)- BaF_2_ (1.8 μm)).

Supplementary figure 5 shows the absorption as a function of incident wavelength and angle for thermal emitter. It is evident from Supplementary figure 6 that by increasing the incident angle, the absorption is reducing significantly at higher angles.


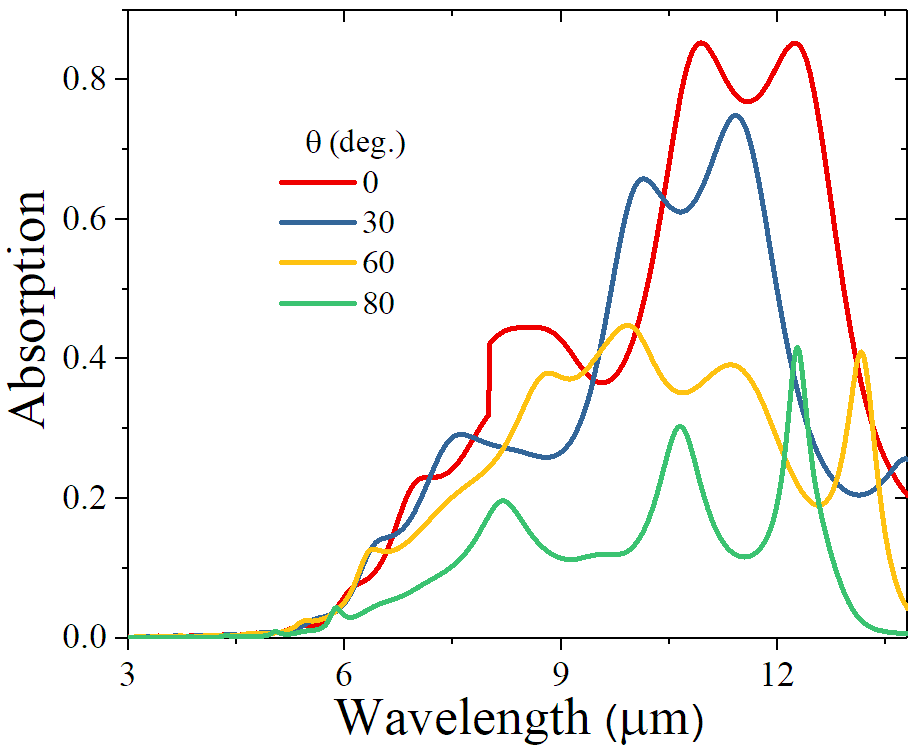


**Supplementary Figure 5.** Absorption as a function of incident angle and wavelength

**Fabrication Tolerance of the proposed Thermal Emitter**

Finally, we examine the effects of fabrication errors on the designed emitter. The simulation is done for absorption as a function of wavelength and the % change in the thickness of each layer with respect to the optimized thickness at four angles 0 deg, 20 deg, 40 deg, and 60 deg, as shown in figure 6. The white horizontal line represents the emitter with optimized thicknesses that is 1.8 μm, 2.9 μm, 0.1 μm, 3 μm,1.8 μm, 2.9 μm, 1.8 μm, 0.5 μm, 0.8 μm, 0.5 μm, respectively for BaF_2_, LiF, BaF_2_, LiF, BaF_2_, LiF, BaF_2_, LiF, Si3N4 and LiF, respectively. Considering thickness errors in thin film deposition system which can be up to 5 %, we vary the thickness of each layer in emitter from -5 % to +5 %. That means we calculate the absorption by simply increasing and decreasing the thicknesses of each layer in the emitter up to 5 %. The region below the white dotted line represents negative thickness error down to -5 %. In all cases with increasing or decreasing the thicknesses of all layers in an emitter, the resonance wavelengths remain the same, with insignificant variation in absorption.


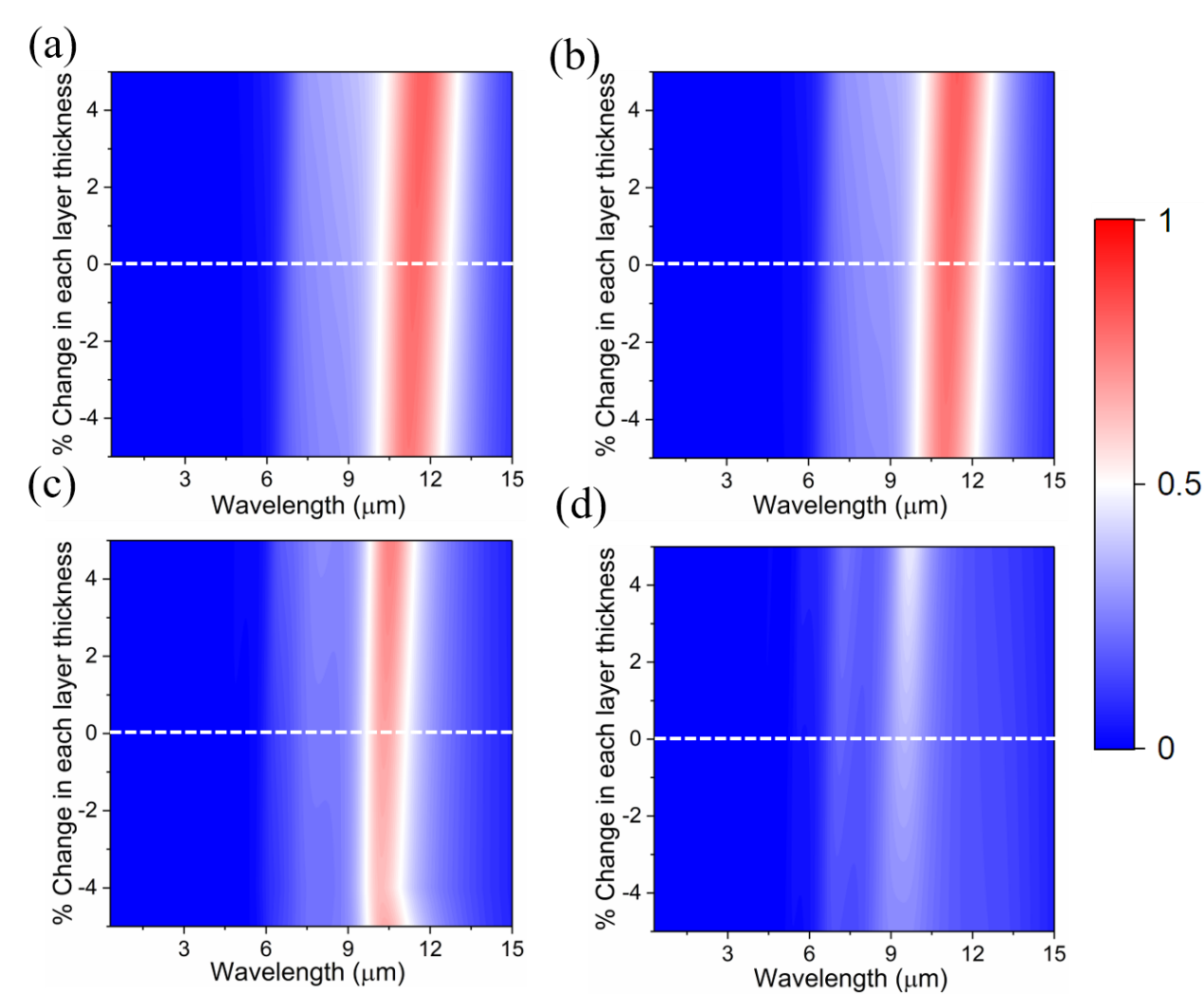


**Supplementary Figure 6.** Absorption as a function of wavelength and % thickness change from the optimized thicknesses of each layer at incident angle of (a) 0 deg, (b) 20 deg, (c) 40 deg and (d) 60 deg. A design with optimized parameters is shown by a white dotted horizontal line.

In Figure 7, the variation of absorption is shown as a function of incident angle at actual emitter thickness (0 %), at 5 % increment in thickness of each layer of emitter (+ 5%) and 5% decrements of thickness of each layer of emitter (- 5%) with respect to 0%. The change in absorption < $\pm4\%$. It is noteworthy that the number of layers involved in our proposed angularly selective thermal emitter is orders of magnitude less than in previous reports^9–11^ and with such an excellent fabrication tolerance a practical device can be designed and manufactured with such excellent performance.


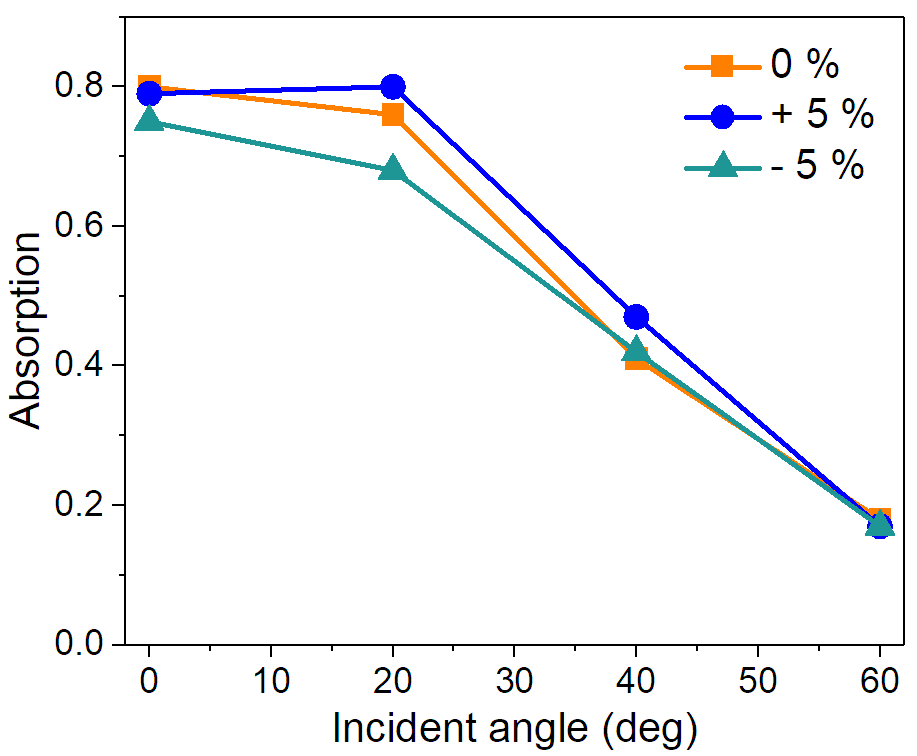


**Supplementary Figure 7.** Absorption as a function of incident angle at wavelength of 11 $\mu m$. Data is taken from figure 6.

**Transient Analysis**

The time to reach a steady state temperature can be calculated using following equation ^12^.

$$AP_{net}+mC\frac{dT}{dt}=0$$

Where A is the area of cooler, m is the mass and C is the specific heat capacity. Considering a cooler area of $30 cm \times30 cm$, a mass m calculated using the volume and density of each material and the corresponding C for each layer. The net power Pnet when the temperature of the cooler and ambience are the same, is 50 W/m2. Pnet is calculated using the energy balance equation 1 in the main manuscript and iterated with time evolution. We also consider conduction and convection losses with a parasitic heat transfer coefficient of h= 0.2 ${Wm}^{-2}K^{-1}$. As an extreme case, we consider a long event where the atmospheric absorptivity/emissivity is 1, i.e., the sky becomes completely opaque to IR radiation for an extended period sufficient to heat up the radiative cooler back to ambient temperature (~ 45 minutes) as shown in the green shaded region. After the event ends and the atmospheric emissivity is back to its original value (~ 0.2) the emitter cools down to its subfreezing temperature in ~90 mins steady state.


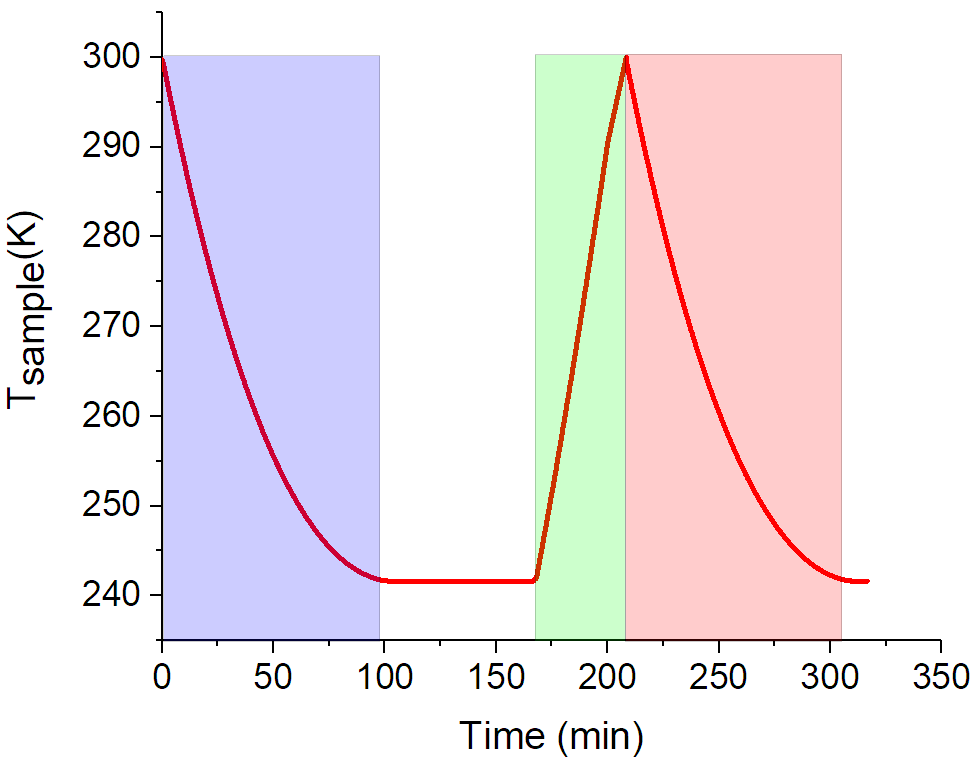


**Supplementary Figure 8.** Steady state time and cooler temperature for angularly selective system.

On the other hand, if we reduce the thermal emission selectivity by only allowing spectral selectivity, the equilibrium time is much faster. For a spectrally (but not angularly) selective thermal emitter, the emitter reaches steady state temperature after ~ 50 minutes as shown below.


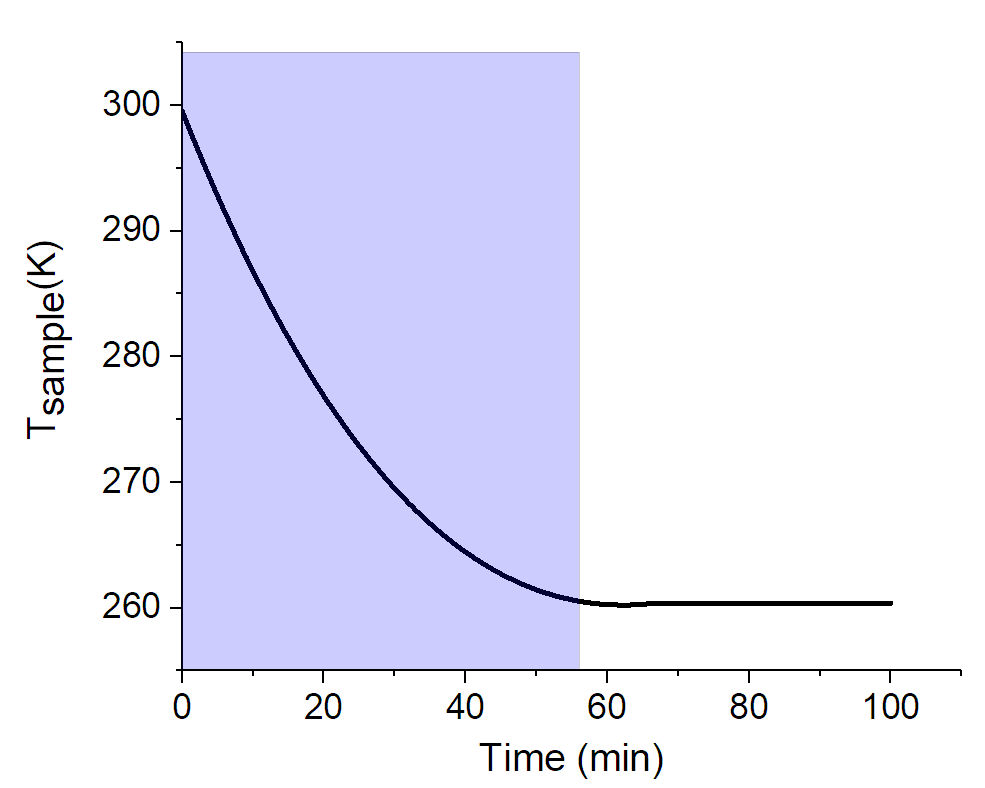


**Supplementary Figure 9.** Steady state time and cooler temperature for only spectrally selective system.

These results are in general agreement with experimental studies by Chen et. al.^13^.

**References:**

1. Moore, H. H. Refractive index of alkaline earth halides and its wavelength and temperature derivatives. *J. Phys. Chem. Ref. Data* **9**, 161–290 (1982).

2. Yokota, H. *et al.* Broadband mid-infrared frequency comb generation in a Si3N4 microresonator. *Opt. Lett.* **40**, 4823–4826 (2015).

3. Kischkat, J. *et al.* Mid-infrared optical properties of thin films of aluminum oxide, titanium dioxide, silicon dioxide, aluminum nitride, and silicon nitride. *Appl. Opt.* **51**, 6789–6798 (2012).

4. Zhao, Dongliang, Ablimit Aili, Yao Zhai, Shaoyu Xu, Gang Tan, 4 Xiaobo Yin and Ronggui Yang. Radiative sky cooling : Fundamental principles , materials , and applications. *Appl. Phys. Rev.* **6**, 021306 (2019).

5. ElKabbash, M. *et al.* Iridescence-free and narrowband perfect light absorption in critically coupled metal high-index dielectric cavities. *Opt. Lett.* **42**, 3598 (2017).

6. Bathgate, S. N. & Bosi, S. G. A robust convection cover material for selective radiative cooling applications. *Sol. Energy Mater. Sol. Cells* **95**, 2778–2785 (2011).

7. Debenham, M. Refractive indices of zinc sulfide in the 0405–13-μm wavelength range. *Appl. Opt.* **23**, 2238 (1984).

8. Heavens, O. S. *Thin-film Optical Filters*. *Optica Acta: International Journal of Optics* vol. 33 (CRC press, 1986).

9. Qu, Y. *et al.* Polarization-Independent Optical Broadband Angular Selectivity. *ACS Photonics* **5**, 4125–4131 (2018).

10. Qu, Y., Pan, M. & Qiu, M. Directional and spectral control of thermal emission and its application in radiative cooling and infrared light sources. *Phys. Rev. Appl.* **13**, 1 (2020).

11. Shen, Y. *et al.* Optical broadband angular selectivity. *Science (80-. ).* **343**, 1499–1501 (2014).

12. Aili, A., Yin, X. & Yang, R. Passive sub-ambient cooling: radiative cooling versus evaporative cooling. *Appl. Therm. Eng.* **202**, 117909 (2022).

13. Chen, Z., Zhu, L., Raman, A. & Fan, S. Radiative cooling to deep sub-freezing temperatures through a 24-h day-night cycle. *Nat. Commun.* **7**, 1–5 (2016).
